# On the reliability of motor evoked potentials in hand muscles of healthy adults: a systematic review

**DOI:** 10.3389/fnhum.2023.1237712

**Published:** 2023-08-31

**Authors:** Mirja Osnabruegge, Carolina Kanig, Florian Schwitzgebel, Karsten Litschel, Wolfgang Seiberl, Wolfgang Mack, Martin Schecklmann, Stefan Schoisswohl

**Affiliations:** ^1^Institute of Psychology, University of the Bundeswehr Munich, Neubiberg, Germany; ^2^Department of Psychiatry and Psychotherapy, University of Regensburg, Regensburg, Germany; ^3^Department of Electrical Engineering, University of the Bundeswehr Munich, Neubiberg, Germany; ^4^Institute of Sport Science, University of the Bundeswehr Munich, Neubiberg, Germany

**Keywords:** transcranial magnetic stimulation, motor evoked potentials, reliability, primary motor cortex, hand muscles, healthy humans, systematic review

## Abstract

**Aims:**

Motor evoked potentials (MEP) elicited by transcranial magnetic stimulation (TMS) over the primary motor cortex are used as a neurophysiological marker of cortical excitability in clinical and scientific practice. Though, the reliability of this outcome parameter has not been clarified. Using a systematic approach, this work reviews and critically appraises studies on the reliability of MEP outcome parameters derived from hand muscles of healthy subjects and gives a proposal for most reliable TMS practice.

**Methods:**

A systematic literature research was performed in PubMed, according to the PRISMA guidelines. Articles published up to March 2023 that were written in English, conducted repeated measurements from hand muscles of healthy subjects and reliability analysis were included. The risk of publication bias was determined. Two authors conducted the literature search and rated the articles in terms of eligibility and methodological criteria with standardized instruments. Frequencies of the checklist criteria were calculated and inter-rater reliability of the rating procedure was determined. Reliability and stimulation parameters were extracted and summarized in a structured way to conclude best-practice recommendation for reliable measurements.

**Results:**

A total of 28 articles were included in the systematic review. Critical appraisal of the studies revealed methodological heterogeneity and partly contradictory results regarding the reliability of outcome parameters. Inter-rater reliability of the rating procedure was almost perfect nor was there indication of publication bias. Identified studies were grouped based on the parameter investigated: number of applied stimuli, stimulation intensity, reliability of input-output curve parameters, target muscle or hemisphere, inter-trial interval, coil type or navigation and waveform.

**Conclusion:**

The methodology of studies on TMS is still subject to heterogeneity, which could contribute to the partly contradictory results. According to the current knowledge, reliability of the outcome parameters can be increased by adjusting the experimental setup. Reliability of single pulse MEP measurement could be optimized by using (1) at least five stimuli per session, (2) a minimum of 110% resting motor threshold as stimulation intensity, (3) a minimum of 4 s inter-trial interval and increasing the interval up to 20 s, (4) a figure-of-eight coil and (5) a monophasic waveform. MEPs can be reliably operationalized.

## 1. Introduction

Since the introduction of transcranial magnetic stimulation (TMS) by Barker et al. (1985), the majority of studies use this non-invasive brain stimulation technique to stimulate the primary motor cortex (M1) in order to provoke a quantifiable response of the human motor system. Applied over M1, single TMS pulses elicit motor evoked potentials (MEPs) that can be recorded via electromyography (EMG) from the corresponding contralateral target muscle. MEPs are frequently used as physiological markers of corticospinal excitability (CSE) in scientific research and clinical practice (Rossini et al., 2015). The electrical stimulation of the M1 evokes a complex pattern of early direct pyramidal tract axon activation and later indirect activation of axonal connections (Di Lazzaro and Rothwell, 2014). Activation is triggered due to potential changes along the propagation of the axon, resulting primarily in an activation pattern of axons perpendicular to the induced current flow in the brain (Di Lazzaro and Rothwell, 2014).

Motor evoked potentials are most often derived from the subject’s hand muscles via surface electrodes attached to the target muscle of interest. Commonly used outcome measures of M1 stimulation are the contralateral derived peak-to-peak amplitude, the area under the curve (AUC) which is defined as the integral of the rectified signal and the input-output curve (IO-curve or stimulus-response curve). While the amplitude (MEP_amp_) is a direct measure of CSE, the IO-curve represents the amplitude as a function of the stimulation intensity (Devanne et al., 1997; Rossini et al., 2015). For the quantification of these outcome measurements, the amplitude signal of EMG responses is necessary. However, it was shown that the amplitude exhibits high intrinsic variability. This variability could be attributed to spontaneous intra-individual changes and fluctuations in CSE (Kiers et al., 1993; Rossini et al., 2015), and is present even at the same level of stimulation intensity (Kiers et al., 1993; Wassermann, 2002). Inter-individual anatomical differences (Pellegrini et al., 2018a) and technical stimulation parameters could contribute to this variability as well. For example, the use of different waveforms, coil-types and the orientation of the coil cause different current flows in the cortex. This in turn leads to varied patterns of focality, neuronal population activation and recruitment (Kiers et al., 1993; Di Lazzaro et al., 2004; Di Lazzaro and Rothwell, 2014; Rossini et al., 2015).

The number of annually published studies using TMS to elicit MEPs is growing steadily. Without sufficient clarification about the reliability of outcome measurements, valid interpretations of available findings are rather limited. Besides validity and objectivity, reliability is one of the main quality criteria requirements for high-quality and standardized research. Reliability is statistically described as the ratio of the variance of the true value to the overall variance. A reliable measurement instrument produces consistent values with low measurement error. As the observed value always consists of the true value and the inseparable measurement error, reliability rather describes an estimation of the error. Or, vice versa, the degree to which the measurement is free from error. Ultimately, the reliable instrument is thus able to distinguish true changes in the target variable from random or systematic errors (Atkinson and Nevill, 1998; Bialocerkowski and Bragge, 2008; Mokkink et al., 2010; Portney and Watkins, 2015). Portney and Watkins (2015) argue that with identifying the factors that affect the observed values, more variance can be predicted and the amount of unaccounted variance attributed to error decreased. To be successfully used in research and clinical practice, e.g., as a diagnostic instrument, the assessment of MEPs must be reliable. The categorization of whether a variable or instrument is reliable or not depends on the one hand on the inherent characteristics of the variable, and on the other hand on the appraisal of the reliability coefficient by the experimenter. The experimenter must decide which reliability coefficient value is suitable on the basis of the knowledge about the target variable (Portney and Watkins, 2015).

However, at this stage no review has systematically addressed the reliability of single pulse MEP-measurements in healthy subjects. Thus, this systematic review aims to identify studies reporting on the reliability of MEPs evoked via single TMS-pulses and derived from relaxed hand muscles of healthy individuals. The main objective of the present review is to not only give an overview about the available studies addressing the reliability of MEPs, but also to reach a conclusion about the reliability of MEP-measurements as well as to identify stimulation parameters that potentially produce most reliable MEP measurements. These will be combined into a best-practice recommendation for the reliable detection of single-pulse MEPs. For this purpose, the MEP amplitude, AUC and IO-curves as stimulation outcome measures together with the corresponding statistical reliability parameters are extracted per study for reliability evaluation. In order to be able to assess the quality of the individual studies with regard to the experimental procedure and to increase transparency, a critical assessment of the quality and methodology of the articles is carried out by two authors using a standardized evaluation scale, namely, Chipchase et al.’s (2012) Checklist.

## 2. Methods

### 2.1. Literature research

A systematic literature search was conducted in March 2023 according to the Preferred Reporting System for Reviews and Meta-Analysis (PRISMA) guidelines (Page et al., 2021). All articles published up to that time were considered for further assessment. The publication date of the earliest included study was 2001, and that of the most recent was 2022. The literature search was performed using the keywords and the Medical Subjects Headings thesaurus (MeSH-terms) of the National Library of Medicine indexing PubMed articles “transcranial magnetic stimulation” and “motor evoked potential” or “MEPs” or “cortical excitability” and “reliability” or “repeatability” or “reproducibility” in PubMed. In addition, the reference sections of the resulting single studies were screened for further applicable papers. Two independent authors (MO and CK) conducted the literature search separately as well as rated the found articles with respect to eligibility: In a first step, the titles and abstracts of the entries were screened whether they were addressing the corresponding topic. In a second step, the articles thus classified as suitable were examined in full-text form with respect to the inclusion and exclusion criteria as outlined in the following section.

### 2.2. Inclusion- and exclusion criteria

Studies were classified as eligible if they met the following inclusion criteria: (1) TMS application in healthy adult subjects; (2) derivation of MEPs from hand muscles; (3) written in English; (4) conducted repeated measures, respectively proper reliability analysis (test-retest, intra- or inter-rater reliability); (5) report of at least one statistical reliability parameter.

**FIGURE 1:**
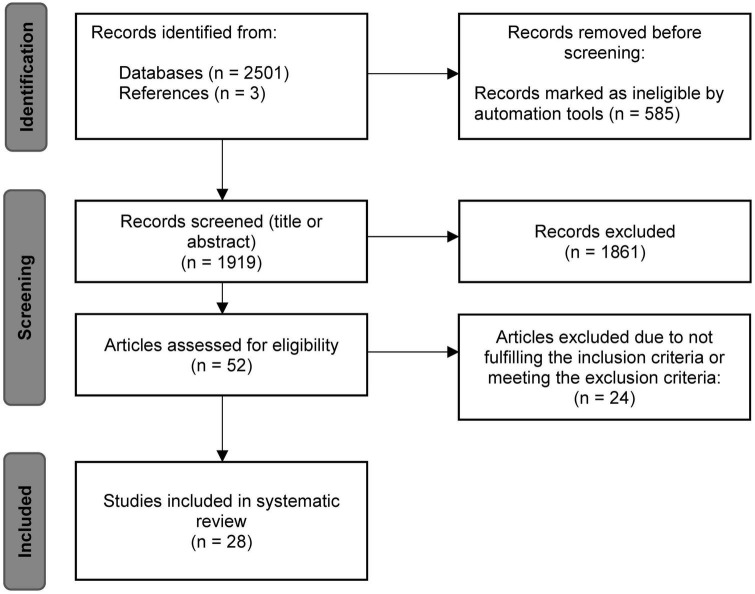
Flowchart of the systematic literature search based on the PRISMA statement (Page et al., 2021).

Not included were (1) other reviews, single case or single trial studies, study protocols or comments, studies that investigated (2) animal models or (3) participants under the age of 18 years or (4) lower limb or arm muscles and (5) papers not written in English. Figure 1 shows the literature search process according to the PRISMA guidelines (Page et al., 2021). A total number of 2,501 entries could be identified using the above-mentioned search string in PubMed. Three additional studies were found, screened and included based on the references of the PubMed articles. Of the 2,501 records, 585 were removed by automatic search filters, i.e., human subjects, ≥18 years. During the screening of titles and abstracts, a further 1,861 articles were sorted out individually by both raters. The remaining 52 entries were reviewed in detail for meeting or not meeting the inclusion and exclusion criteria. At the end of the process, a total number of 28 studies were identified as eligible and included in the present review.

### 2.3. Study and reliability assessment

After article eligibility evaluation, the data on the (1) subject characteristics, (2) stimulators and coils used, (3) stimulation intensity, (4) target muscle, (5) waveform as well as the (6) number of sessions, (7) time interval between measurements, (8) applied stimuli, (9) TMS outcome parameters, (10) their reliability parameters, and (11) intervals between measurements were extracted from the final 28 articles and summarized by the first author. Next, the two raters (MO and CK) assessed the studies independently regarding the fulfilment of items in the checklist of Chipchase et al. (2012) which is described in detail in the next section. The inter-rater reliability of the checklist rating was determined via calculation of Cohen’s kappa (Cohen, 1960). Absolute and relative frequencies of the criteria fulfilment were determined study- and item-wise. A total score was calculated by adding the number of fulfilled criteria and dividing by the total number of applicable criteria per study. The method of checklist application and inter-rater reliability calculation was conducted following Beaulieu et al. (2017).

In order to further check for possible publication bias, a funnel plot was used (Light and Pillemer, 1984) and tested for asymmetry with linear regression after the method of Egger et al. (1997). As described, a rigid classification of reliability coefficients is not recommended and existing limits are arbitrary (Portney and Watkins, 2015). In order to give an orientation about the existing values, reliability coefficients below 0.50 are described as poor, between 0.50 and 0.75 as moderate and above 0.75 as good (Portney and Watkins, 2015), while the value 1.00 would indicate perfect reliability.

### 2.4. Chipchase et al.’s Checklist

Given the growing number of TMS studies of the human motor system and the variability in outcome measures, Chipchase et al. (2012) designed a checklist to assess the methodological quality of studies with the goal of increasing data quality in this research field. The checklist consists of 30 items which allow a critical evaluation of the reported methodology (Chipchase et al., 2012). As we were not interested in paired-pulse paradigms, the items concerning these and the unconditioned MEP size, were excluded from the rating and analysis. The items assessing the use of medication (i.e., use of CNS active drugs and prescribed medication) were combined. The checklist was completed under the following assumptions:

In the scope of the checklist, the gender is not a variable that would necessarily be important to control and extent of relaxation of muscle other than those being tested is not a reportable factor. In the present review, these items and other items were assessed as controllable, i.e., when the sample was gender-balanced and reportable, i.e., if the activation level of other muscles was monitored. The sole statement that a procedure (e.g., determination of resting motor threshold, RMT) was carried out was not sufficient to evaluate the item as reported - this only applied if the used method was mentioned. If variables were balanced (e.g., gender balance), controlled via e.g., a questionnaire or included as a factor in the statistical analysis, they were rated as controlled. An item that is considered as controlled will also be rated as reported. Since the term gender was used in the checklist, this term refers to the sex of the studied subjects and is retained to avoid further complexity. Results regarding the checklist and inter-rater reliability can be found in the Supplementary material.

### 2.5. Association of checklist criteria or reliability values with publication date

Based on the hypothesis that scientific and technological progress in the scientific field increases with time, Pearson correlation was calculated in SPSS (V29.0, IBM Corp., USA) to test whether there is an association between the number of fulfilled checklist criteria or reliability values with ongoing publication year.

## 3. Results

The included studies as well as subject characteristics, stimulators and coils used, stimulation intensity, target muscle and waveform are shown in Table 1. In summary, 588 subjects with an average age of 32 ± 6 years were examined in the studies, of which 247 (42%) were female participants. Regarding the different muscles of the hand, the majority of the studies examined the first dorsal interosseus muscle (FDI) (*n* = 23), followed by the abductor pollicis brevis (APB) (*n* = 5) and abductor digiti minimi (ADM) (*n* = 1). In total, 19 studies used a device from Magstim^®^, six from MagVenture^®^ and two studies each used a device from Cadwell^®^ or NexStim^®^. Most frequently, a figure-of-eight (Fof8) coil was used for stimulation (*n* = 22, of which one was angulated), five studies used a circular coil and in three cases the coil type was not defined. The range of stimulation intensities used in the studies ranged from 90 to 170% RMT, respectively 5–100% of the maximum stimulator output (MSO). Two studies used a stimulation intensity that elicits a MEP_amp_ of approximately 1 mV (SI_1mV_). The majority of the experiments was conducted with a monophasic (*n* = 17) or a biphasic (*n* = 5) waveform, whilst two studies applied both waveforms. In the remaining four studies, the used pulse shape could not be clearly identified. For 13 studies the waveform is derived e.g., from the description of the current flow in the respective studies marked with asterisks in Table 1. Note that some studies used multiple stimulators, different coil types and waveforms or compared more than one muscle.

**TABLE 1:** Participant characteristics and TMS parameters.

References	Participants	Age	TMS-device and coil-type	Stimulation intensity	Target muscle	Waveform
		M ± SD, range		%MSO,%RMT, SI_1mV_		
Bashir et al., 2017	20 (*f* = 6)	23.9 ± 2.9	MagPro X100 n.a.	120% RMT	FDI	monophasic*
Bastani and Jaberzadeh, 2012	12 (*f* = 6)	30.3 ± 6.8	Magstim 200^2^ Fof8	120% RMT	FDI	monophasic*
Biabani et al., 2018	15 (*f* = 8)	21.5 ± 3.1	MagPro R30 angulated Fof8	SI_1mV_	FDI	biphasic
Brown et al., 2017	84 (*f* = n.a.)	n.a.	Magstim 200^2^ Fof8	110%, 130%, 150% RMT	APB	monophasic
Carroll et al., 2001	8 (*f* = 0)	22 – 36	Magstim 200 Fof8	–5, 0, 5, 10, 15, 20, 30, 40% MSO at RMT, 100% MSO	FDI	monophasic*
Chang et al., 2016	54 (*f* = 24)	61.7 ± 13.1	Magstim Super Rapid n.a.	120% RMT	FDI	biphasic
Christie et al., 2007	30 (*f* = 15)	76 ± 6.3	Cadwell MES-10 Circular coil	110%, 130%, 150% RMT	ADM	n.a.
Cueva et al., 2016	20 (*f* = n.a.)	18 – 86	MagPro X100 Circular coil	120%, 140% RMT	FDI	biphasic
Cuypers et al., 2014	36 (*f* = 18)	20.5 ± 1.2	Magstim BiStim^2^ Fof8	110%, 120% RMT	FDI	monophasic*
Davila-Pérez et al., 2018	23 (*f* = 13)	18–35	MagPro X100 Fof8	120% RMT	FDI	mono- and biphasic
Dyke et al., 2018	10 (*f* = 7)	24 ± 4	Magstim BiStim Fof8	100, 110, 120, 130, 140, 150% RMT	FDI**	monophasic**
Fleming et al., 2012	10 (*f* = 3)	26 – 61	Magstim 200^2^ Fof8 and circular	90, 100, 110, 120, 130% RMT	FDI	monophasic*
Goldsworthy et al., 2016	47 (*f* = 21)	24.6 ± 4.6	Magstim 200 Fof8	120%, 150% RMT	FDI	monophasic
Hashemirad et al., 2017	23 (*f* = 21)	25.3 ± 6.8	Magstim 2002, MagPro R30 Fof8	120% RMT, SI_1mV_	FDI	monophasic*
Hassanzahraee et al., 2019	15 (*f* = 9)	24.1 ± 5.4	MagVenture n.a. Fof8	120% RMT	FDI	biphasic
Julkunen et al., 2009	8 (*f* = 4)	n.a.	eXimia Fof8	120% RMT	APB	biphasic
Jung et al., 2010	8 (*f* = 4)	23.8 ± 1.2	Magstim 200 Fof8	5% steps between –5 and 130% RMT	APB	monophasic*
Kamen, 2004	14 (*f* = 5)	24.4 ± 8.2	Cadwell MES-10 Focal coil	70%, 85%, 100% MSO	FDI	n.a.
Kukke et al., 2014	10 (*f* = 5)	28.0 ± 8.0	Magstim 200^2^ Fof8	5% increments between 5 and 100% MSO	FDI	monophasic
Liu and Au-Yeung, 2014	14 (*f* = 9)	27.4 ± 3.4	Magstim Fof8	100%, 110%, 120%, 130%, 150% RMT	FDI	n.a.
Malcolm et al., 2006	20 (*f* = 10)	26.9 ± 4.5	Magstim Rapid, Fof8 and Magstim 200, circular	5% steps between 30 and 100% MSO	APB FDI	bi- (Fof8) and monophasic (circular coil)
McDonnell et al., 2004	10 (*f* = 5)	19–38	Magstim 200 Circular coil	110%, 120% RMT	FDI	monophasic*
Ngomo et al., 2012	12 (*f* = 7)	26.0 ± 4.3	Magstim BiStim Fof8	110%, 120% RMT	FDI	monophasic*
Nguyen et al., 2019	9 (*f* = 6)	21–29	NexStim eXimia Fof8	120% RMT	FDI	n.a.
Pellegrini et al., 2018b	12 (*f* = 6)	29.3 ± 2.8	Magstim Fof8	105%, 120%, 135%, 150%, 165% RMT	FDI	monophasic*
Schambra et al., 2015	21 (*f* = 11)	64.7 ± 10.1	Magstim BiStim^2^ Fof8	100%, 110%, 130%, 150%, 170% RMT	FDI	monophasic*
Therrien-Blanchet et al., 2022	31 (*f* = 18)	23.1 ± 3.6	Magstim 200^2^ Fof8	100%, 110%, 120%, 130%, 140% RMT	APB	monophasic*
Vaseghi et al., 2015	12 (*f* = 6)	32.3 ± 7.2	Magstim 2002 Fof8	120% RMT	FDI	monophasic*

Included studies and their methodological parameters. ADM, abductor digiti minimi; APB, abductor pollicis brevis; FDI, first dorsal interosseous; f, female participants; Fof8, figure of eight coil; MSO, maximum stimulator output; n.a., not available; RMT, resting motor threshold; SI_1mV_, intensity that evokes MEPs of ∼1 mV; *, the information is not clearly stated in the original study but derived from the reported parameters, e.g., stimulator handbook or electrode model, current flow in posterior-anterior current direction or **stated in another referred study.

### 3.1. TMS and reliability measurement within the studies

Table 2 shows the TMS outcome parameters, the number of sessions, time interval between measurements and applied stimuli and the statistical reliability indices of the individual studies. These were grouped according to the variable for which reliability was determined: Number of applied stimuli, stimulation intensity (SI), target muscle or target hemisphere, reliability of IO-curve parameters, inter-trial interval (ITI), current direction, coil type and use of neuronavigation systems, used for precise positioning of the coil relative to the brain. The listing of studies in multiple categories is possible.

**TABLE 2:** TMS and reliability measurement within the studies.

References	TMS outcome	Number of Stimuli, sessions, and intervals			Statistical reliability index
	AUC, MEP_amp_, IO-curve	Number of (per session) applied stimuli	Minutes (min), days (d), weeks (w), months (m)	Short-/Long-term, Within-/Between-sessions	CI, CV, IC, ICC, κ
**Number of applied stimuli**					
Bashir et al., 2017	MEP_running average amp_	2 × 40	2 sessions, 1 w	Long-term, between-session	*N* = 31–40 pulses have 100% chance of inclusion in the 95% CI_40_^a^
Bastani and Jaberzadeh, 2012	MEP_amp_	3 × 15 1 × 15	20 min (T_1_-T_2_-T_3_) 2 sessions, ≥48 h (T_1_-T_4_)	Short- and long-term, within- and between-session	ICC_within–session, 5, 10, 15 trials_ = 0.93, 0.98, 0.98 ICC_between–session, 5, 10, 15 trials_ = 0.88, 0.93, 0.93
Biabani et al., 2018	MEP_amp_ MEP_running average amp_	2 × 35 1 × 35	20 min (T_1_-T_2)_ 2 sessions, 1 w (T_1_-T_3_)	Short- and long-term, within- and between-session	ICC_within–session, 5, 15–35 trials_ = *0.13*, 0.8 ICC_between–session, 5, 20_ = *0.16*, 0.78
					*N* = 19 pulses have 100% chance of inclusion in the 95% CI_35_^b^
Chang et al., 2016	MEP_amp_ MEP_running average amp_	1 × 30	1 session	Within-session	*N* = 17 pulses have 90% chance of inclusion in the 95% CI_30_^b^ *N* = 20 pulses 95% chance *N* = 21 pulses 100% chance
Christie et al., 2007	MEP_amp_	1 × 10 per intensity in blocks of 2, 3, 4, 5 trials	1 session	Within-session	ICC_2,3 trials_ = poor^c^ ICC_4 trials_ = > 0.70 ICC_5 trials_ = ≥ 0.90
Cuypers et al., 2014	MEP_amp_ MEP_running average amp_	2 × 40	1 session, 2 min	Short-term, within-session	*N* = 26–29 pulses have 99% chance of inclusion in the 95% CI_40_ *N* = 30–40 pulses 100%
Goldsworthy et al., 2016	MEP_running average amp_	2 × 40	25 min 2 sessions, 14–33 d	Within-session	*N* = 15 pulses have 62% chance of inclusion in the 95% CI_37_ *N* = 20 pulses 87% *N* = 29 pulses 100%
				Short-term, within-session	ICC_trial 1–5_ = poor^d^ ICC_trial 6–10_ = fair ICC_trial 11–15, 16–20_ = good ICC_trial 21–25, 26–30, 31–35_ = excellent
				Long-term, between-session	ICC_trial 1–5_ = poor ICC_trial 6–10_ = poor ICC_trial 11–15, 16–20, 21–25_ = fair ICC_trial 26–30, 31–35_ = good
Hashemirad et al., 2017	MEP_amp_	2 × 20 1 × 20	20 min 2 sessions, ≥72 h	Short- and long-term, between-session	ICC_120% MT; trial 1–10_ = 0.851 ICC_120% MT; trial 1–15_ = 0.897 ICC_120% MT; trial 1–20_ = 0.922 ICC_SI1mV%; trial 1–10_ = 0.533 ICC_SI1mV%; trial 1–15_ = 0.721 ICC_SI1mV%; trial 1–20_ = 0.770
Nguyen et al., 2019	MEP_amp_	1 × 150	1 session	Within-session	20 trials held approx. 90% total variance of full dataset
**Stimulation intensity**					
Brown et al., 2017	MEP_amp_ AUC	n.a.	3 sessions, 3 study sites, 12 m	Long-term, between-session	ICC_110% MT_ < 0.40 ICC_130% MT_ = 0.70 ICC_150% MT_ = 0.81 ICC_AUC 110% MT_ < 0.40 ICC_AUC 130% MT_ = 0.60 ICC_AUC 150% MT_ = 0.52
Christie et al., 2007	MEP_amp_	1 × 10 per intensity, use of first 5 trials per block	2 sessions, 20 min	Short-term, between-sessions	ICC_110% MT_ = 0.83 ICC_130% MT_ = 0.65 ICC_150% MT_ = 0.82
Cueva et al., 2016	MEP_amp_ IO-curve	n.a.	2 session, 30 min	Short-term, between-sessions intra- and inter-rater	Cronbach’s α_120% MT_ = 0.489 Cronbach’s α_140% MT =_ 0.803 κ_120% MT_ = 0.575 κ_140% MT_ = 0.851
Cuypers et al., 2014	MEP_running average amp_	2 × 40	2 min	Short-term	N_110% MT_ = ≥ 26 stimuli have 100% chance of inclusion in the 95% CI_40_ N_120% MT_ = ≥ 30 stimuli
Kamen, 2004	MEP_amp_	3 × 10 in blocks of 5 stimuli	3 sessions, ≥48 h	Long-term, between-session	ICC_70% MSO_ = 0.81 ICC_85% MSO_ = 0.75 ICC_100% MSO_ = 0.60
Ngomo et al., 2012	MEP_amp_	n.a.	3 sessions, 4 d, 35-457 d^e^	Long-term (T_1_-T_2,_ T_1_–T_3_), between-session	ICC_110% T1–T2_ = 0.70 ICC_120% T1–T2_ = 0.87 ICC_110% T1–T3_ = 0.20 ICC_120% T1–T3_ = 0.75
Pellegrini et al., 2018b	MEP_amp_	5 × 25	3 sessions, 20 min (T_1_-T_2_), ≥48 h (mean 7.25 d) (T_1_-T_3_)	Short- and long-term, within- and between session	ICC_within–session, 105, 120, 135, 150, 165% MT_ = *0.568*, 0.717^f^, 0.900^f^, 0.968^f^, 0.923^f^ ICC_between–session, 105, 120, 135, 150, 165% MT_ = *0.333*, 0.660, 0.686, 0.789^f^, 0.860^f^
**Target muscle or hemisphere**					
Malcolm et al., 2006	IO-curve	15 × 5	2 sessions, 14 d	Long-term, between-session	ICC_APB IO–slope_ = 0.78 ICC_FDI IO–slope_ = 0.82
McDonnell et al., 2004	MEP_amp_ AUC	2 × 20	<1 h	Short-term, within-session	ICC_FDI_ = 0.46–0.55 > ICC_FCU_
Schambra et al., 2015	IO-curve	2 × 10	4 sessions, 3.5-5 h (T_1_-T_2_, T_3_-T_4_), 24 h (T_2_-T_3_)	Short- and long-term, between-session	ICC_left–hemisphere MEPmax_ = 0.90 ICC_left–hemisphere s50_ = 0.91 ICC_left–hemisphere IO–slope_ = 0.03 ICC_right–hemisphere MEPmax_ = 0.82 ICC_right–hemisphere s50_ = 0.92 ICC_right–hemisphere IO–slope_ = 0.07
**IO-curve**					
Carroll et al., 2001	IO-curve	9 × 10 in blocks of 2 × 3 and 1 × 4 stimuli	2 min 3 sessions, ≥24 h	Short- and long-term, within- and between-session	ICC_within–session; MEPmax_ = 0.60 ICC_within–session; s50/peak slope_ = 0.63 ICC_within–session; IO–slope_ = 0.77 ICC_between–session; MEPmax_ = 0.82 ICC_between–session; s50/peak slope_ = 0.84 ICC_between–session; IO–slope_ = 0.91
Dyke et al., 2018	IO-curve	6 × 10	2 blocks (181.8 d) of 4 sessions (3-4 d)	Long-term, between-session	ICC_IO–slope, all–sessions_ = 0.807 ICC_IO–slope, block 1_ = 0.923 ICC_IO–slope, block 2_ = 0.862
Kukke et al., 2014	IO-curve	2 × 20	2 sessions, 15 min	Short-term, within-session	ICC_MEPmax_ = 0.94 ICC_s50_ = 0.84 ICC_IO–slope_ = 0.60
Liu and Au-Yeung, 2014	IO-curve	5 × 10	2 sessions, 7 d	Long-term, between-session	ICC_IO–slope_ = 0.75 ICC_MEPmax_ = 0.87
Therrien-Blanchet et al., 2022	IO-curve	5 × 10	4 sessions, ≥48 h	Long-term, between-session	ICC_IO–total–slope_ = 0.76 ICC_IO–mid–slope_ = 0.64 ICC_IO–end–slope_ = 0.57
**Inter-trial Interval**					
Hassanzahraee et al., 2019	MEP_amp_	4 × 25	20 min 2 sessions, ≥ 48 h	Short- and long-term, within- and between-session	ICC_within–session; ITI 5, 10, 15, 20s_ = 0.79, 0.86, 0.89, 0.90 ICC_between–session; ITI 5, 10, 15, 20s_ = 0.79, 0.83, 0.86, 0.89
Vaseghi et al., 2015	MEP_amp_	2 × 15 per ITI	4 sessions, 20 min (T_1_-T_2_-T_3_-T_4_), 2 sessions ≥ 48 h (T_1_-T_5_)	Short- and long-term, within- and between-session	ICC_ITI 4s within–session_ = 0.96 ICC_ITI 10s within–session_ = 0.95 ICC_ITI 4s between–session_ = 0.87 ICC_ITI 10s between–session_ = 0.80
**Current direction and waveform**					
Davila-Pérez et al., 2018	MEP_amp_	1 × 10	2 sessions, 1–70 d	Long-term, between-session	ICC_monoAP_ = 0.69 ICC_monoPA_ = 0.56 ICC_biAP–PA_ = −0.16
**Coil Type and Navigation**					
Fleming et al., 2012	MEP_amp_ IO-curve	1 × 10 per intensity	3 sessions, average 7 day (3–14 d)	Long-term, between-session	ICC_Fof8 120%_ = 0.75 ICC_circular coil 120%_ = 0.09 ICC_navigated Fof8 120%_ = 0.55 ICC_Fof8 MEPsum_ = 0.81 ICC_circular coil MEPsum_ = 0.48 ICC_navigated Fof8 MEPsum_ = 0.80
Julkunen et al., 2009	MEP_amp_	2 × 20	2 sessions, 2–7 d	Long-term, between-session	CV_navigated_ = 71 ± 14%< CV_non–navigated_ = 91 ± 15%
Jung et al., 2010	IO-curve	1 × 20 per intensity	3 sessions, ≥24 h	Long-term, between-session	CV_navigated_ = CV_non–navigated_

The table shows the outcome parameters, the number of trials and sessions as well as the statistical indices used within the studies. The number of applied stimuli and between-session interval columns read as follows: e.g., (Bastani and Jaberzadeh, 2012) conducted two sessions, within session one they applied 3 blocks of 15 consecutive stimuli; these blocks were separated by a 20-min break. After a period of at least 48 h they conducted the second session in which they applied solely 15 stimuli. Within-session values refer to the values calculated between T_1_-T_2_-T_3_, the blocks of 15 stimuli applied within the first session. Between-session values refer to the calculations between T_1_ and T_4_. Comparisons between outcome measures within a day are categorized as short-term, comparisons with an interval ≥ 24 h are categorized as long-term reliability. Cursive values indicate non-significant statistics, if not otherwise stated *α* = 0.05.

^a^31–40 pulses yield a probability of 100% that the true MEP_amp_ is included in the 95% CI calculated on running average MEP_amp_.

^b^95% CI around the mean MEP amplitude.

^c^except for 110% MT, ICC classification after Atkinson and Nevill (1998).

^d^poor (<0.40), fair (0.40–0.58), good (0.59–0.75), or excellent (>0.75) (Cicchetti and Sparrow, 1981).

^e^ range 35–457 days, median interval 88 days.

^f^p < 0.01; AUC, area under the curve; CI, confidence interval; CV, coefficient of variation; FDI, first dorsal interosseus; FCU, flexor carpi ulnaris; ICC, Intraclass correlation coefficient; IC, internal consistency, Cronbach’s alpha; IO-curve, input-output curve/recruitment curve; IO-slope, slope of the IO-curve; ITI, inter-trial interval, synonymous inter-stimulus interval; κ, Cohen’s Kappa; MEP_amp_, amplitude of the motor evoked potential; MEP_max_, plateau of the IO-curve; MEP_running average amp_, running average of the MEP_amp_; MT, (resting) Motor threshold; MSO, maximum of stimulator output; n.a., not applicable, i.e., information not stated in the article; Peak slope, peak slope that occurs at the stimulus intensity equal to s_50_; s_50_, stimulus intensity that evokes a MEP size halfway between the baseline and plateau.

Nine of the identified studies investigated the effect of the number of applied stimuli on the reliability of the MEP_amp_ (Christie et al., 2007; Bastani and Jaberzadeh, 2012; Goldsworthy et al., 2016; Hashemirad et al., 2017; Biabani et al., 2018) or the probability of inclusion of the running average of MEP_amp_ - the average calculated on consecutive trials - in the 95% confidence interval of all trials (CI_n_-method) (Cuypers et al., 2014; Chang et al., 2016; Goldsworthy et al., 2016; Bashir et al., 2017; Biabani et al., 2018). One study used a principal component regression approach to determine the number of trials and the corresponding amount of variance that is accounted for by them (Nguyen et al., 2019).

Seven studies investigated the effects of stimulation intensity on reliability (Kamen, 2004; Christie et al., 2007; Ngomo et al., 2012; Cueva et al., 2016; Brown et al., 2017; Pellegrini et al., 2018b) as well as the inter-rater reliability (Cohen’s κ) and internal consistency (Cronbach’s α) of the ratings (Cueva et al., 2016) and the influence of intensity in the scope of the CI method (Cuypers et al., 2014).

The majority of the identified studies chose the FDI as the target muscle for MEP derivation, two studies performed a direct comparison with other hand or forearm muscles in an experiment (McDonnell et al., 2004; Malcolm et al., 2006).

Reliability of the different IO-curve parameters (slope, peak-slope and maximum/plateau) were also investigated by five studies (Carroll et al., 2001; Kukke et al., 2014; Liu and Au-Yeung, 2014; Dyke et al., 2018; Therrien-Blanchet et al., 2022) and compared between the contra- and ipsilateral sides within one study (Schambra et al., 2015).

The influence of the length of the ITI was investigated by two studies (Vaseghi et al., 2015; Hassanzahraee et al., 2019), the influence of current direction by one study (Davila-Pérez et al., 2018) and the influence of the used coil (Fof8 coil vs. circular coil) was directly compared within one study (Fleming et al., 2012). Two direct comparisons between the measurement with and without the use of navigation were made based on coefficient of variation (CV) values (Julkunen et al., 2009; Jung et al., 2010).

### 3.2. Identifying best practice

Comparing the results of the identified studies, recommendations regarding the reliable estimation, i.e., a high ratio of true variance to overall variance, of CSE parameters (MEP_amp_, IO-curve, AUC) with single-pulse TMS can be derived.

With a minimum of 19 and a maximum of 31 pulses an estimation with 100% chance of inclusion in the running average 95% CI of the intra- (Cuypers et al., 2014; Chang et al., 2016; Goldsworthy et al., 2016; Biabani et al., 2018) and inter-session (Bashir et al., 2017) amplitude is possible. One study used the CI-method to compare the inclusion probability between 110 and 120% RMT stimulation intensity and reported an attainment of 100% inclusion in the CI after 26 pulses at the lower stimulation intensity and after 30 pulses at the higher intensity (Cuypers et al., 2014).

The reliability values for the minimum number of trials required within- and between a session are heterogeneous. While three studies report “poor” values for five or less trials (Christie et al., 2007; Goldsworthy et al., 2016; Biabani et al., 2018) within a session, one study reported moderate reliability for four (Brown et al., 2017) and two studies good values for five trials (Christie et al., 2007; Bastani and Jaberzadeh, 2012).

For between-session comparisons, values were also “poor” [<0.40 (Goldsworthy et al., 2016);0.16 (Biabani et al., 2018)] for five or less trials in two studies, but also classified as good (0.88) within one study (Bastani and Jaberzadeh, 2012).

Applying six to 15 trials within a session resulted in “fair” (0.40–0.58 after Cicchetti and Sparrow, 1981) reliability in one study (Goldsworthy et al., 2016), while almost perfect values of ICC = 0.98 were reached after 10 trials within one study. This value did not increase further when increasing the number from 10 to 15 trials (Bastani and Jaberzadeh, 2012).

In between-session comparisons, one study reported “poor” (<0.40) values for six to 10 trials (Goldsworthy et al., 2016). Almost good [>0.851 (Hashemirad et al., 2017)] to perfect values [ = 0.93, (Bastani and Jaberzadeh, 2012)] were reported applying 10 to 15 trials by two studies, while one reported “fair” [0.40–0.58 (Goldsworthy et al., 2016)] values for 11 to 15 trials.

Increasing the number of applied stimuli within a session further was done within one study, resulting in “good” (0.59–0.75 after Cicchetti and Sparrow, 1981) reliability for 16 to 20 trials, while after the 21st pulse up to 35 pulses “excellent” (>0.75) values were reported (Goldsworthy et al., 2016).

Between-sessions, “fair” [0.40–0.58 (Goldsworthy et al., 2016)] values were reported for 16 to 25 trials by one study, with increasing values up to “good” (0.59–0.75) reliability after 26 to 35 trials (Goldsworthy et al., 2016); while in another study good reliability values for amplitudes were reached after applying 15 and up to 35 trials (Biabani et al., 2018). For SI_1mV_, between-session reliability values linearly increased from the moderate values within the first 10 and 15 applied stimuli to good values applying 20 stimuli (Hashemirad et al., 2017).

This heterogeneous pattern of results regarding the optimal number of applied stimuli within and between sessions does not allow an unambiguous statement. However, the values suggest that a minimum number of five stimuli within and between sessions should be applied per intensity for reliable measurement. Furthermore, at least a trend of increasing ICC values with the number of stimuli seems reasonable, as shown for SI_1mV_ (Hashemirad et al., 2017).

The stimulation intensity shows a heterogeneous pattern as well. One study reports a decrease in reliability with increasing stimulation intensity (Kamen, 2004). However, in addition to a finding with high reliability at lowest and highest intensity and a decrease at medium intensity (u-shape) (Christie et al., 2007), four studies show higher reliability with increasing stimulation intensity within- and between-sessions (Ngomo et al., 2012; Cueva et al., 2016; Brown et al., 2017; Pellegrini et al., 2018b). On a descriptive basis, the use of a stimulation intensity ≥ 110% RMT could produce more reliable results.

Reliability of the parameters of the IO-curve (MEP_max_, slope and s_50_ - the amplitude that evokes a MEP halfway between baseline and MEP_max)_ were estimated within seven studies (Carroll et al., 2001; Malcolm et al., 2006; Kukke et al., 2014; Liu and Au-Yeung, 2014; Schambra et al., 2015; Dyke et al., 2018; Therrien-Blanchet et al., 2022). ICC values for the slope were classified as good in five studies (Carroll et al., 2001; McDonnell et al., 2004; Malcolm et al., 2006; Liu and Au-Yeung, 2014; Therrien-Blanchet et al., 2022) and moderate in one (Kukke et al., 2014). Peak slope ICC values and maxima were classified as good in three (Carroll et al., 2001; Kukke et al., 2014; Schambra et al., 2015) respective four studies (Carroll et al., 2001; Kukke et al., 2014; Liu and Au-Yeung, 2014; Schambra et al., 2015) and moderate in one each (Carroll et al., 2001). Only in one study investigating older adults, the reliability of the slope was classified as poor (Schambra et al., 2015). In that study, a comparison of IO-curve parameters collected in both hemispheres from primarily right-handed elderly subjects showed good ICC values for s_50_ and plateau in both hemispheres, whereas the values for the slope were poor (ICC < 0.07). Therefore, reliable derivation of s_50_ and plateau is possible in both the right and left hemisphere, i.e., bilateral (Schambra et al., 2015).

Prolonging the time-interval from 5, 10, 15 to 20 s between single pulse applications further increases good intra- and inter-session reliability values in one experiment (Hassanzahraee et al., 2019). In contrast, another study directly comparing ITIs of 4 s and 10 s did not show an increasing reliability with increasing ITI. In this case, intra- and inter-session reliability was good for 4 s as well as 10 s intervals (Vaseghi et al., 2015). A reliable estimation of MEP amplitude with a minimum ITI of 4 s therefore is possible and an increase of up to 20 s could further increase reliability.

In addition, the use of a Fof8 coil was superior in terms of between-session reliability to the use of a circular coil, also under the benefit of navigation (Fleming et al., 2012). Regarding the comparison between the applied pulse shape, the use of a monophasic waveform was more beneficial in reliably estimating amplitude than a biphasic waveform (Davila-Pérez et al., 2018).

The contradictory nature of the results on the influence of navigation on the CV does not yet allow a statement to be made at this point in time (Julkunen et al., 2009; Jung et al., 2010).

### 3.3. Publication bias

**FIGURE 2:**
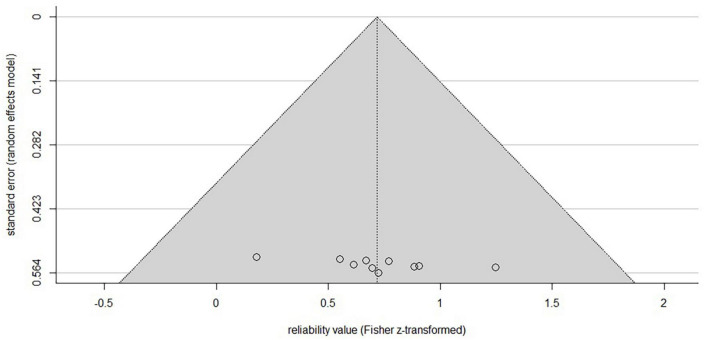
Funnel plot of the included studies reporting ICC-values. *X*-axis is showing the z-transformed mean ICC-values, the *y*-axis the standard error. The horizontal line indicates the population effect size, skewed lines the 95% CI. Although the typical inverted funnel shape is not evident, a significant symmetrical distribution centered at the bottom indicates the typical small sample sizes (higher standard error) in non-invasive brain studies with low risk of publication bias. Note that studies reporting other parameters than ICC are not included.

A funnel plot (Figure 2) was created in R (R Core Team, Austria, V. 4.0.5; Schwarzer et al., 2015) to check for publication bias within studies reporting ICC-values (Light and Pillemer, 1984). The linear regression approach to test for asymmetry after Egger et al. (1997) revealed no significant asymmetric distribution (*t* = 2.12, df = 8, *p* = 0.067), indicating that included studies are not subject to publication bias. As standard error decreases with increasing sample size it would theoretically reach zero with infinite sample size. It can be seen from the distribution of the single study values at the bottom of the plot, that all studies deploy a small sample size as indicated by relatively higher standard errors, as typical for non-invasive brain stimulation studies.

## 4. Discussion

The present review work identified studies on the reliability of MEPs evoked via single TMS-pulses and derived from relaxed hand muscles of healthy individuals. It aims to give an overview of the available studies addressing the reliability of MEPs and to identify technical TMS parameters that produce most reliable MEP measurements. For this purpose, a systematic literature search up to March 2023 was conducted, according to the PRISMA guidelines (Page et al., 2021). A total of 28 articles addressing the research topic were identified and most relevant parameters were descriptively summarized. The identified studies were assigned to seven different categories and the results are discussed in detail: number of applied stimuli (*n* = 9 studies); stimulation intensity (*n* = 7); target muscle or hemisphere (*n* = 3); IO-curve (*n* = 6); ITI (*n* = 2); waveform and current direction (*n* = 1); coil type and navigation (*n* = 3).

### 4.1. Number of applied stimuli

According to the CI method, the 100% probability of inclusion in the 95% of the respective studies was achieved for 19–31 stimuli (Cuypers et al., 2014; Chang et al., 2016; Goldsworthy et al., 2016; Bashir et al., 2017; Biabani et al., 2018). For reliable detection of amplitude within and between sessions, at least five stimuli should be applied, whereby higher ICC values are also reported with an increasing number of stimuli (Christie et al., 2007; Bastani and Jaberzadeh, 2012; Goldsworthy et al., 2016; Hashemirad et al., 2017; Biabani et al., 2018). In their calculations, Nguyen et al. (2019) described that 20 stimuli within a session held circa 90% of the total variance of the dataset. Based on reliability theory, the true MEP amplitude cannot be measured, as all measured values contain inseparable systematic or random measurement errors. Thus, to judge what degree of reliability is sufficient for the measured variable is strongly based on the nature of the variable itself and evaluation of the experimenter. Therefore, following reliability theory and considering amplitude variability, the best approximate estimate of true MEP_amp_ can be achieved by averaging single trials (Portney and Watkins, 2015; Rossini et al., 2015). As systematic errors are constant and make up a smaller proportion of the total error than random error, they rather impact validity than reliability. Therefore, with averaging trials, the random errors arising from e.g., unknown technical interfering in the laboratory, could cancel each other out (Portney and Watkins, 2015). These assumptions lead to the question of how many individual stimuli should be applied during a session, which is always a trade-off between time and accuracy. Ammann et al. (2020) addressed the question of the optimal number of stimuli per session in their theoretical and experimental framework. Their results support the assumption that an exact optimal number of pulses is not generalizable for such a highly intrinsic-variable outcome parameter as the MEP_amp_. Rather, the assumption of reliability theory must also be considered here as to what extent of error variance is assumed to be reasonable for the experiment and variable. Furthermore, their analytical results suggest that the optimal number of stimuli needed for reliable MEP amplitude estimation is dependent on the total number of applied stimuli, and the more stimuli are applied in total the more are needed for a suitable estimation (Ammann et al., 2020). Thus, the analytical results of Ammann et al. (2020) are a limiting factor in the generalizability of the studies investigating the optimal number of stimuli. However, they provide support for the observation in the included studies of our review that at a certain number of stimuli, a plateau effect occurs (which seems to occur between 19 and 31 stimuli in the studies examined here) at which reliability does not appear to increase further.

### 4.2. Stimulation intensity

While in one case lower reliability values are described with increasing stimulation intensity and in another case a u-shaped course is reported, four studies show a linear increase (Kamen, 2004; Christie et al., 2007; Ngomo et al., 2012; Cueva et al., 2016; Brown et al., 2017; Pellegrini et al., 2018b). The majority of results regarding higher stimulation intensities and increasing reliability values are to be expected due to the underlying corticospinal processes: with an increasing stimulation intensity, the MEP_amp_ increases due to a faster and uniform recruitment of the underlying neural connections and corticospinal fibers (Rossini et al., 2015), which could reflect in the positive linear relationship of increased reliability and lowered variability at higher stimulator output. As the stimulation intensity and MEP amplitude increase, a plateau is reached from which the CSE does not increase further, partially based on the rising phase cancellation of the underlying motor unit action potentials (Rossini et al., 2015). This is also partly observable for the reliability values at higher intensities. For example, between two sessions, the reliability continues to rise with an increase from 150 to 165% RMT and remains in the upper category within a session from 135% RMT on to 165% (Pellegrini et al., 2018b). However, this does not explain the results of decreasing reliability values with increasing stimulation intensity, which were reported in two studies (Kamen, 2004; Christie et al., 2007). One possible explanation is the heterogeneity of the technical experimental parameters used (e.g., other stimulator and coil type), which are described further below.

### 4.3. Maximum stimulator output

One problem of comparability between different TMS studies is the parameter Maximum Stimulator Output to which the used stimulation intensity is mostly relativized to. This indicates the stimulator-specific generated output and is not transferable to other stimulators due to different manufacturers and models, which makes results comparability more difficult. In order to still be able to achieve a replicability of the stimulation dose, Peterchev et al. (2012) recommend reporting all parameters that have an influence on the induced electromagnetic field (i.e., stimulation device, settings, coil type and waveform parameters e.g., pulse width, ITI).

### 4.4. Inter-trial and inter-session interval

With increasing the ITI up to 20 s, variability could be reduced; amplitude - and in one case reliability - could be increased (Kamen, 2004; Hassanzahraee et al., 2019). The underlying mechanisms are not yet fully understood, but the authors attribute them to suprathreshold post-stimulus change of hemodynamic processes that take a certain amount of time to return to baseline levels. For example, after suprathreshold stimulation of the prefrontal cortex, the level of oxy-hemoglobin decreases, reaching a minimum at circa 8 s post-stimulus (Kamen, 2004; Thomson et al., 2011, 2012; Hassanzahraee et al., 2019). As the studies did not compare ITI shorter than 4 s, assumption about shorter time-intervals can not be made. Results regarding the underlying hemodynamic processes would suggest that a further reduction of ITI might not be beneficial.

The inter-session interval within the studies was grouped in short-term (≤24 h) and long term (≥24 h) intervals. Descriptively, no trend of higher reliability with short- or long intervals can be derived.

### 4.5. Current direction

One study showed that applying pulses with a monophasic waveform resulted in higher reliability than compared to a biphasic waveform, regardless of the direction of induced current flow in the cortex (Davila-Pérez et al., 2018). Both the waveform of a pulse applied with a controllable pulse TMS (cTMS) and the induced current flow in the motor cortex affect the motor threshold, MEP latency and steepness of the IO-curve in the FDI at rest (Sommer et al., 2018). In the cTMS study is described that a symmetrical biphasic pulse can be viewed as two monophasic pulses with opposite directions, which result in the activation of distinct directional specific neuronal populations (Sommer et al., 2018). At this point, our literature search identified one study (Davila-Pérez et al., 2018) dedicated to the reliability of CSE parameters with different pulse shapes and current directions. The authors suggest that the successive components of the biphasic pulse lead to a cancellation of activation due to simultaneous activation of inhibitory and excitatory neuronal circuits (Davila-Pérez et al., 2018). Thus, the inconsistent activation pattern at the investigated stimulation intensity could have led to the low reliability for the biphasic in comparison to the monophasic waveform.

At this point it is important to highlight the general differences between the stimulator manufacturers, complicating the comparison of the output of the devices already described. As Schoisswohl et al. (2023) highlighted in their comparison of current directions in the repetitive TMS treatment of tinnitus disorder, the default current direction of TMS-devices varies between fabricators. The manufacturer differences also relate to the winding of the coils and nomenclature of the current direction in the coil. Also surprising in this context is that the majority of the included studies used a monophasic waveform for stimulation, as primarily biphasic pulses are used for repetitive TMS-treatment (Rossini et al., 2015).

### 4.6. Coil-type

Using a Fof8 coil was superior in terms of between-session reliability to the use of a circular coil, regardless of whether neuronavigation was used. The Fof8 coil with its two interfering electric and magnetic fields induces higher currents directly under the coil than in the periphery, whilst the circular coil induces a steady circular current flow under the coil (Di Lazzaro and Rothwell, 2014). Stimulation with circular coils tends to be less focal than stimulation with Fof8 coils and results in higher descending output when applied above motor threshold intensity. This higher output of non-focal stimulation in the form of spinal volleys could be due to the more widespread activation that can also occur on the non-targeted hemisphere. Further, it is possible that the direction of the induced current under the round coil in the brain tissue is more inhomogeneous than the current flow generated by a Fof8 coil (Di Lazzaro et al., 2004). It is therefore obvious that despite the use of the same stimulation parameters, different corticospinal excitation patterns are produced when using the two types of coils (Di Lazzaro et al., 2004) and comparability of studies using distinct coil models is further limited. Future studies should explicitly investigate the reliability of different coil types and also include new coil designs.

### 4.7. Use of navigation

Surprisingly, the results of the direct comparisons of the influence of navigation on the CV are contrary. The CV as a measure of outcome parameter stability decreased significantly when navigation was used for MEP_amp_ measurement in one study (Julkunen et al., 2009), but remained unchanged in another study measuring IO-curves (Jung et al., 2010). These results are counterintuitive, as coil positioning stability is increased when neuronavigation is used (Cincotta et al., 2010). Jung et al. (2010) state that they controlled other sources of MEP variability e.g., coil orientation, coil type, electrode placement and level of target muscle relaxation. Therefore, the authors propose the result of comparable CV values measured with and without the use of navigation in their findings to be caused by the spontaneous fluctuations of CSE, as described earlier. In contrast, Julkunen et al. (2009) interpret the observed higher and more stable MEP amplitudes as a result of higher stimulation precision, leading to a more efficient recruitment of neurons. As a result, intra-individual variation decreased (Julkunen et al., 2009).

### 4.8. Target muscle or hemisphere

Studies in which the result parameters were directly compared explicitly in different hand muscles are still rare, but one study showed comparable good reliability values in the two hand muscles (Malcolm et al., 2006), while the slope of the IO-curve in FDI scored slightly higher ICC-values than the APB (ICC 0.82 > 0.78). Comparison with upper extremity muscles within the study showed comparable or lower ICC values for muscles of the forearm. However, in a direct comparison of the FDI with forearm muscle Flexor carpi ulnaris, ICC values of amplitude were classified as poor to moderate but higher than in the forearm (McDonnell et al., 2004). Future studies should aim for a reliability comparison of MEP measures across single target muscles.

### 4.9. IO-curve

The IO-curve represents the amplitude as a function of the stimulation intensity, which follows a sigmoidal shape that can be described by the slope, the intensity that evokes a response half the size of the maximum amplitude and the maximal amplitude respective plateau (Rossini et al., 2015). Except for one study describing poor reliability for the slope parameter, the other five studies show moderate to good slope reliability. Overall, the IO-curve can be used to reliably measure CSE in healthy humans.

### 4.10. Reliability coefficients and statistical parameters

The most common outcome parameter for determining reliability within the studies examined was the ICC, which ranged from −0.16 for biphasic pulses between sessions (Davila-Pérez et al., 2018) to 0.98 for 15 trials applied with monophasic pulses within a session (Bastani and Jaberzadeh, 2012). As the coefficient is calculated on the basis of intra-subject and sample variances, which certainly differ between samples, the comparison between studies is generally difficult and results can only be extrapolated to resembling samples. The second most frequently reported measure of outcome parameter stability was CV, which is a relative, unit-free measure that allows for comparability between studies (Portney and Watkins, 2015). In terms of ICC, reporting the exact model that was used for calculation (Koo and Li, 2016) and all relevant experimental parameters is an approach to increase transparency and comparability in the research field. Like the MEP_amp_ itself is highly variable, so are the studies examining it. The problem of inconsistent results does not only concern studies on single pulses, but has also been described for widely used repetitive neuromodulatory TMS-protocols and repetitive heuristics (Prei et al., 2023). One approach to increase transparency and decrease inconsistency of results can be the use of standardized checklists.

### 4.11. Chipchase et al.’s Checklist and inter-rater agreement

To increase transparency, the studies were evaluated by two raters with regard to their methodological criteria using the standardized checklist by Chipchase et al. (2012). Inter-rater reliability of the checklist rating was calculated. Furthermore, the hypothesis that the number of fulfilled checklist criteria also increases with progressing publication year, due to advances in technology and research, was tested via Pearson correlation. Contrary to expectations, no association was found between publication date and checklist score. On average, the relative sum of reported and controlled items per study reached 46.8% and 17 of the 28 rated studies reached a total score of ≥ 50%. Mean inter-rater reliability, expressed by Cohen’s Kappa, was 0.87. This value describes an almost perfect agreement (Landis and Koch, 1977) between the two authors who rated the studies independently. Detailed results can be found in the Supplementary material. Future studies should use the checklist for orientation and report the listed parameters in order to increase the interpretability of individual studies and transparency within the research field. The development of a standardized scale for categorizing the results could increase comparability.

### 4.12. Identifying best practice

For reliable measurement it can be beneficial to use: (1) at least five stimuli per session, (2) a minimum of 110% RMT as stimulation intensity, (3) a minimum of 4 s ITI and increasing the ITI up to 20 s, (4) a figure-of-eight coil, and (5) a monophasic waveform.

### 4.13. Limitations

This systematic review is limited by several factors e.g., the specific focus on those studies that have investigated the reliability of single parameters, thus, generalizable statements about possible interactions are not applicable. Furthermore, despite a careful literature search and selection of criteria, it cannot be disproven that relevant articles were not included. Since a meta-analytic summary was not appropriate due to the number and structure of the available data, the results are on a descriptive basis and as outlined in the discussion, interpretation should be done with caution. Further studies should target the topic of reliability in comprehensive designs. e.g., targeting the interaction of stimulation intensity, number of pulses and pulse shape. Lastly, this study only investigated studies of hand muscles of healthy individuals, and it is therefore unclear whether these results can be extrapolated to other muscle groups or clinical populations. Computational models and simulations are necessary to include multiple parameters and evaluate their interactional impact in the future.

## 5. Conclusion

This systematic review aimed to give an overview about studies reporting on the reliability of MEPs evoked via single TMS-pulses in relaxed hand muscles of healthy adults. It gives a summary statement of the reliability scores as well as identified technical parameters and their influence on these reliability values. Parameters that could contribute to more reliable outcome measures can be descriptively identified. For reliable measurement it can be beneficial to use: (1) at least five stimuli per session, (2) a minimum of 110% RMT as stimulation intensity, (3) a minimum of 4 s ITI and increasing the ITI up to 20 s, (4) a figure-of-eight coil, and (5) a monophasic waveform. MEPs can be reliably derived and expressed with MEP_amp_, AUC and IO-curve from the hand muscles of healthy subjects. Future studies are needed to investigate reliability in clinical populations and in experimental designs examining factor interactions.

## Data availability statement

The original contributions presented in this study are included in the article/Supplementary material, further inquiries can be directed to the corresponding author.

## Author contributions

MO did the conceptualization of methodology, literature search, rating, visualization, analysis, and wrote the original draft. CK did literature search, rating, visualization, analysis, reviewed, and edited the manuscript. FS, KL, WS, WM, and MS reviewed and edited the manuscript. SS did the supervision, conceptualization of methodology and writing, and reviewed and edited the manuscript. All authors read and approved the final manuscript.
