# Supplementary material for: On the reliability of motor evoked potentials in hand muscles of healthy adults: a systematic review
Source: Front Hum Neurosci. 2023 Aug 31;17:1237712. doi: 10.3389/fnhum.2023.1237712 (PMC10500067; doi:10.3389/fnhum.2023.1237712)
Supplement: Supplementary file 1 [file Table_1.docx]

Supplementary Material

On the Reliability of Motor Evoked Potentials in Hand Muscles of Healthy Adults: A Systematic Review

**Mirja Osnabruegge^1,2*^, Carolina Kanig^1,2^, Florian Schwitzgebel^3^, Karsten Litschel^3^, Wolfgang Seiberl^4^, Wolfgang Mack^1^, Martin Schecklmann^2^, Stefan Schoisswohl^1,2^**

^1^Institute of Psychology, University of the Bundeswehr Munich, Neubiberg, Germany.

^2^Department of Psychiatry and Psychotherapy, University of Regensburg, Regensburg, Germany.

^3^Department of Electrical Engineering, University of the Bundeswehr Munich, Neubiberg, Germany.

^4^Institute of Sport Science, University of the Bundeswehr Munich, Neubiberg, Germany.

*** Correspondence:**

Mirja Osnabruegge
mirja.osnabruegge@unibw.de

# Supplementary Results

## Chipchase rating frequencies & Inter-rater agreement

During the critical evaluation of 28 studies with the standardized checklist of Chipchase et al. (2012), a heterogeneity in the methods of the studies was found. This makes it difficult - in combination with the heterogeneity of statistical reliability and technical parameters - to compare the results of the individual subcategories. The absolute frequencies of either reported or controlled items of the Chipchase et al.’s checklist are depicted in the supplementary **Table S3** as well as the inter-rater agreement of the ratings expressed via Cohen´s Kappa and corresponding confidence intervals in the supplementary **Table S4**. A mean amount of 68.3% (range 3.6-100%) of all items was rated as reported and a mean of 25% (range 0-61%) as controlled. The item with the lowest total score, therefore which was rated the least as reported or controlled, was the level of relaxation of muscles other than those being tested (1.8%). The item stimulation intensity had the highest total score (78.6%).

The mean relative sum of reported plus controlled items per study was 46.8% (29.2-74%) of the total applicable items. Of all 28 identified studies, 17 reached a value of ≥ 50%. At this point, no ordinal scale exists to put the checklist scores in relation. Since there is no classification or comparison of the number of fulfilled checklist criteria, an interpretation of the results is not possible and the results are merely descriptive. Cohen’s Kappa agreement ranged from −0.23, which can be classified as poor, to an almost perfect correlation of 1.00 (mean 𝜅 = .87) (Landis and Koch, 1977). The average 𝜅-value indicates a high degree of agreement, thus increasing the transparency and objectivity of the rating procedure.

Due to technological development and research advancements, it was assumed that more criteria of the Chipchase checklist are met, the later the study was published. No significant correlation was evident between the publication year and the number of fulfilled Chipchase’ criteria (*r* = .052, *p =* .794*)* or between publication year and mean intra-class correlation coefficient (ICC) values (*r* = .054, *p =* .808). Due to the lack of significant correlations, no further analyses were carried out.

**Supplementary Table S3**

*Absolute & relative frequencies of the Chipchase’ items*

|  | **Reported**  **(n/N)** | | **Controlled**  **(n/N)** | | **Total**  **(%)** | |
| --- | --- | --- | --- | --- | --- | --- |
| **Participant factors** | | | | | | |
| Age of subjects | | 27/28 | | 2/28 | | 51.8% |
| Gender of subjects | | 28/28 | | 14/28 | | 75.0% |
| Handedness of subjects | | 26/28 | | 15/28 | | 73.2% |
| Use of CNS active drugs/prescribed medication | | 9/28 | | 2/28 | | 19.6% |
| Presence of neurological/psychiatric disorders when  studying healthy subjects | | 13/28 | | 2/28 | | 26.8% |
| Any medical condition | | 5/28 | | 2/28 | | 12.5% |
| History of specific repetitive motor activity | | 2/28 | | 0/28 | | 3.6% |
| **Methodological factors** | | | | | | |
| Position and contact of EMG electrodes | | 22/28 | | 6/28 | | 50.0% |
| Amount of relaxation/contraction of target muscles | | 16/28 | | 9/28 | | 44.6% |
| Prior motor activity of the muscle to be tested | | 10/28 | | 10/28 | | 35.7% |
| Level of relaxation of muscles other than those being tested | | 1/28 | | 0/28 | | 1.8% |
| Coil type | | 25/28 | | 1/28 | | 46.4% |
| Coil orientation | | 24/28 | | 2/28 | | 46.4% |
| Direction of induced current in the brain | | 18/28 | | 2/28 | | 35.7% |
| Coil location and stability | | 27/28 | | 13/28 | | 71.4% |
| Type of stimulator used | | 25/28 | | 2/28 | | 48.2% |
| Stimulation intensity | | 28/28 | | 16/28 | | 78.6% |
| Pulse shape (monophasic/biphasic) | | 11/28 | | 2/28 | | 23.2% |
| Determination of optimal hotspot | | 21/28 | | 9/28 | | 53.6% |
| Time between MEP trials | | 26/28 | | 17/28 | | 76.8% |
| Time between days of testing | | 21/21 | | 11/21 | | 76.2% |
| Subject attention (level of arousal) during testing | | 8/28 | | 4/28 | | 21.4% |
| Method for determining threshold | | 25/26 | | 4/26 | | 55.8% |
| Number of MEP measures made | | 24/28 | | 13/28 | | 66.1% |
| **Analytical factors** | | | | | | |
| Method for determining MEP size during analysis | | 27/28 | | 13/28 | | 71.4% |

CNS = central nervous system; EMG = electromyography; MEP = motor evoked potential; n = number of times that the item was rated as reported or controlled; N = number of times that the item was applicable over all studies.

**Supplementary Table S4**

*Inter-rater agreement of Chipchase’ rating per study*

| **Study** | **R -R_1_** | **C-R_1_** | **R-R_2_** | **C-R_2_** | | **Total Score** | **κ** | **Confidence Interval** | | **Limits of Agreement** |  |
| --- | --- | --- | --- | --- | --- | --- | --- | --- | --- | --- | --- |
| [1] | 20 | 5 | 19 | 5 | 50.0 % | | 1.00 | [0.86 | 1.00] | almost perfect | |
| [2] | 19 | 6 | 19 | 4 | 50.0 % | | 0.86 | [0.75 | 0.98] | almost perfect | |
| [3] | 20 | 5 | 18 | 5 | 50.0 % | | 0.78 | [0.64 | 0.92] | substantial | |
| [4] | 17 | 9 | 16 | 7 | 52.0 % | | 0.91 | [0.73 | 1.00] | almost perfect | |
| [5] | 17 | 6 | 17 | 6 | 46.0 % | | 1.00 | [0.86 | 1.00] | almost perfect | |
| [6] | 19 | 5 | 19 | 5 | 50.0 % | | 1.00 | [0.87 | 1.00] | almost perfect | |
| [7] | 15 | 4 | 15 | 4 | 39.6 % | | 1.00 | [0.88 | 1.00] | almost perfect | |
| [8] | 12 | 2 | 12 | 2 | 29.2 % | | 1.00 | [0.87 | 1.00] | almost perfect | |
| [9] | 17 | 6 | 17 | 6 | 47.9 % | | 1.00 | [0.86 | 1.00] | almost perfect | |
| [10] | 21 | 9 | 21 | 9 | 60.0 % | | 1.00 | [0.81 | 1.00] | almost perfect | |
| [11] | 13 | 2 | 10 | 1 | 32.0 % | | -0.11 | [-0.16 | -0.05] | poor | |
| [12] | 18 | 7 | 17 | 7 | 50.0 % | | 0.91 | [0.74 | 1.00] | almost perfect | |
| [13] | 18 | 5 | 18 | 5 | 46.0 % | | 1.00 | [0.87 | 1.00] | almost perfect | |
| [14] | 15 | 3 | 14 | 3 | 36.0 % | | 0.86 | [0.75 | 0.98] | almost perfect | |
| [15] | 19 | 8 | 19 | 8 | 54.0 % | | 1.00 | [0.82 | 1.00] | almost perfect | |
| [16] | 14 | 4 | 14 | 4 | 36.0 % | | 1.00 | [0.90 | 1.00] | almost perfect | |
| [17] | 19 | 7 | 19 | 7 | 52.0 % | | 1.00 | [0.84 | 1.00] | almost perfect | |
| [18] | 13 | 5 | 13 | 4 | 37.5 % | | 0.86 | [0.75 | 0.98] | almost perfect | |
| [19] | 19 | 9 | 18 | 9 | 60.9 % | | 0.91 | [0.72 | 1.00] | almost perfect | |
| [20] | 15 | 2 | 14 | 3 | 34.0 % | | 0.78 | [0.70 | 0.85] | substantial | |
| [21] | 20 | 12 | 20 | 12 | 64.0 % | | 1.00 | [0.80 | 1.00] | almost perfect | |
| [22] | 14 | 6 | 14 | 5 | 40.0 % | | 0.88 | [0.75 | 1.00] | almost perfect | |
| [23] | 13 | 5 | 14 | 5 | 36.0 % | | 0.86 | [0.75 | 0.98] | almost perfect | |
| [24] | 13 | 6 | 13 | 6 | 38.0 % | | 1.00 | [0.85 | 1.00] | almost perfect | |
| [25] | 16 | 7 | 15 | 5 | 46.0 % | | 0.78 | [0.63 | 0.92] | substantial | |
| [26] | 22 | 15 | 20 | 8 | 74.0 % | | 0.40 | [0.27 | 0.53] | fair | |
| [27] | 20 | 6 | 20 | 4 | 54.0 % | | 0.56 | [0.44 | 0.68] | moderate | |
| [28] | 18 | 4 | 18 | 4 | 44.0 % | | 1.00 | [0.89 | 1.00] | almost perfect | |

C-R_1_ = sum of items rated as controlled by rater 1; C-R_2_ = sum of items rated as controlled by rater 2; κ = Cohen´s Kappa; R-R_1_ = sum of items rated as reported by rater 1, R-R_2_ = sum of items rated as reported by rater 2; total score = sum of per study reported or controlled items divided by the sum of applicable items. [1] Bashir et al. (2017); [2] Bastani & Jaberzadeh (2012); [3] Biabani et al. (2018); [4] Brown et al. (2017); [5] Carroll et al. (2001); [6] Chang et al. (2016); [7] Christie et al. (2007); [8] Cueva et al. (2016); [9] Cuypers et al. (2014); [10] Davila-Pérez et al. (2018); [11] Dyke et al. (2018); [12] Fleming et al. (2012); [13] Goldsworthy et al. (2016); [14] Hashemirad et al. (2017); [15] Hassanzahraee et al. (2019); [16] Julkunen et al. (2009); [17] Jung et al. (2010); [18] Kamen (2004); [19] Kukke et al. (2014); [20] Liu & Au-Yeung (2014); [21] Malcolm et al. (2006); [22] McDonnell et al. (2004); [23] Ngomo et al. (2012); [24] Nguyen et al. (2019); [25] Pellegrini et al. (2018b); [26] Schambra et al. (2015); [27] Therrien-Blanchet et al. (2022); [28] Vaseghi et al. (2015).

# References

Bashir, S., Yoo, W.-K., Kim, H. S., Lim, H. S., Rotenberg, A., and Abu Jamea, A. (2017). The Number of Pulses Needed to Measure Corticospinal Excitability by Navigated Transcranial Magnetic Stimulation: Eyes Open vs. Close Condition. Front. Hum. Neurosci. 11, 121. doi: 10.3389/fnhum.2017.00121

Bastani, A., and Jaberzadeh, S. (2012). A higher number of TMS-elicited MEP from a combined hotspot improves intra- and inter-session reliability of the upper limb muscles in healthy individuals. PLoS One 7, e47582. doi: 10.1371/journal.pone.0047582

Biabani, M., Farrell, M., Zoghi, M., Egan, G., and Jaberzadeh, S. (2018). The minimal number of TMS trials required for the reliable assessment of corticospinal excitability, short interval intracortical inhibition, and intracortical facilitation. Neurosci. Lett. 674, 94–100. doi: 10.1016/j.neulet.2018.03.026

Brown, K. E., Lohse, K. R., Mayer, I. M. S., Strigaro, G., Desikan, M., Casula, E. P., et al. (2017). The reliability of commonly used electrophysiology measures. Brain. Stimul*.* 10, 1102–1111. doi: 10.1016/j.brs.2017.07.011

Carroll, T. J., Riek, S., and Carson, R. G. (2001). Reliability of the input–output properties of the cortico-spinal pathway obtained from transcranial magnetic and electrical stimulation. J Neurosci Methods 112, 193–202. doi: 10.1016/S0165-0270(01)00468-X

Chang, W. H., Fried, P. J., Saxena, S., Jannati, A., Gomes-Osman, J., Kim, Y.-H., et al. (2016). Optimal number of pulses as outcome measures of neuronavigated transcranial magnetic stimulation. Clin. Neurophysiol. 127, 2892–2897. doi: 10.1016/j.clinph.2016.04.001

Chipchase, L., Schabrun, S., Cohen, L., Hodges, P., Ridding, M., Rothwell, J., et al. (2012). A checklist for assessing the methodological quality of studies using transcranial magnetic stimulation to study the motor system: an international consensus study. Clin. Neurophysiol. 123, 1698–1704. doi: 10.1016/j.clinph.2012.05.003

Christie, A., Fling, B., Crews, R. T., Mulwitz, L. A., and Kamen, G. (2007). Reliability of motor-evoked potentials in the ADM muscle of older adults. J Neurosci. Methods 164, 320–324. doi: 10.1016/j.jneumeth.2007.05.011

Cohen, J. (1960). A Coefficient of Agreement for Nominal Scales. Educational and Psychological Measurement 20, 37–46.

Cueva, A. S., Galhardoni, R., Cury, R. G., Parravano, D. C., Correa, G., Araujo, H., et al. (2016). Normative data of cortical excitability measurements obtained by transcranial magnetic stimulation in healthy subjects. Neurophysiol. Clin*.* 46, 43–51. doi: 10.1016/j.neucli.2015.12.003

Cuypers, K., Thijs, H., and Meesen, R. L. J. (2014). Optimization of the transcranial magnetic stimulation protocol by defining a reliable estimate for corticospinal excitability. PLoS One 9, e86380. doi: 10.1371/journal.pone.0086380

Davila-Pérez, P., Jannati, A., Fried, P. J., Cudeiro Mazaira, J., and Pascual-Leone, A. (2018). The Effects of Waveform and Current Direction on the Efficacy and Test-Retest Reliability of Transcranial Magnetic Stimulation. Neuroscience 393, 97–109. doi: 10.1016/j.neuroscience.2018.09.044

Dyke, K., Kim, S., Jackson, G. M., and Jackson, S. R. (2018). Reliability of single and paired pulse transcranial magnetic stimulation parameters across eight testing sessions. Brain Stimul. 11, 1393–1394. doi: 10.1016/j.brs.2018.08.008

Fleming, M. K., Sorinola, I. O., Di Newham, J., Roberts-Lewis, S. F., and Bergmann, J. H. M. (2012). The effect of coil type and navigation on the reliability of transcranial magnetic stimulation. IEEE Trans. Neural. Syst. Rehabil. Eng. 20, 617–625. doi: 10.1109/TNSRE.2012.2202692

Goldsworthy, M. R., Hordacre, B., and Ridding, M. C. (2016). Minimum number of trials required for within- and between-session reliability of TMS measures of corticospinal excitability. Neuroscience 320, 205–209. doi: 10.1016/j.neuroscience.2016.02.012

Hashemirad, F., Zoghi, M., Fitzgerald, P. B., and Jaberzadeh, S. (2017). Reliability of Motor Evoked Potentials Induced by Transcranial Magnetic Stimulation: The Effects of Initial Motor Evoked Potentials Removal. Basic Clin. Neurosci. 8, 43–50. doi: 10.15412/J.BCN.03080106

Hassanzahraee, M., Zoghi, M., and Jaberzadeh, S. (2019). Longer Transcranial Magnetic Stimulation Intertrial Interval Increases Size, Reduces Variability, and Improves the Reliability of Motor Evoked Potentials. Brain Connect. 9, 770–776. doi: 10.1089/brain.2019.0714

Julkunen, P., Säisänen, L., Danner, N., Niskanen, E., Hukkanen, T., Mervaala, E., et al. (2009). Comparison of navigated and non-navigated transcranial magnetic stimulation for motor cortex mapping, motor threshold and motor evoked potentials. Neuroimage 44, 790–795. doi: 10.1016/j.neuroimage.2008.09.040

Jung, N. H., Delvendahl, I., Kuhnke, N. G., Hauschke, D., Stolle, S., and Mall, V. (2010). Navigated transcranial magnetic stimulation does not decrease the variability of motor-evoked potentials. Brain Stimul. 3, 87–94. doi: 10.1016/j.brs.2009.10.003

Kamen, G. (2004). Reliability of motor-evoked potentials during resting and active contraction conditions. Med. Sci. Sports Exerc. 36, 1574–1579. doi: 10.1249/01.mss.0000139804.02576.6a

Kukke, S. N., Paine, R. W., Chao, C.-C., Campos, A. C. de, and Hallett, M. (2014). Efficient and reliable characterization of the corticospinal system using transcranial magnetic stimulation. J Clin. Neurophysiol. 31, 246–252. doi: 10.1097/WNP.0000000000000057

Landis, J. R., and Koch, G. G. (1977). The Measurement of Observer Agreement for Categorical Data. Biometrics 33, 159–174.

Liu, H., and Au-Yeung, S. S. Y. (2014). Reliability of transcranial magnetic stimulation induced corticomotor excitability measurements for a hand muscle in healthy and chronic stroke subjects. *J Neurol. Sci.* 341, 105–109. doi: 10.1016/j.jns.2014.04.012

Malcolm, M. P., Triggs, W. J., Light, K. E., Shechtman, O., Khandekar, G., and Gonzalez Rothi, L. J. (2006). Reliability of motor cortex transcranial magnetic stimulation in four muscle representations. Clin. Neurophysiol. 117, 1037–1046. doi: 10.1016/j.clinph.2006.02.005

McDonnell, M. N., Ridding, M. C., and Miles, T. S. (2004). Do alternate methods of analysing motor evoked potentials give comparable results? J Neurosci. Methods 136, 63–67. doi: 10.1016/j.jneumeth.2003.12.020

Ngomo, S., Leonard, G., Moffet, H., and Mercier, C. (2012). Comparison of transcranial magnetic stimulation measures obtained at rest and under active conditions and their reliability. J Neurosci. Methods 205, 65–71. doi: 10.1016/j.jneumeth.2011.12.012

Nguyen, D. T. A., Rissanen, S. M., Julkunen, P., Kallioniemi, E., and Karjalainen, P. A. (2019). Principal Component Regression on Motor Evoked Potential in Single-Pulse Transcranial Magnetic Stimulation. IEEE Trans. Neural. Syst. Rehabil. Eng. 27, 1521–1528. doi: 10.1109/TNSRE.2019.2923724

Pellegrini, M., Zoghi, M., and Jaberzadeh, S. (2018b). The effect of transcranial magnetic stimulation test intensity on the amplitude, variability and reliability of motor evoked potentials. Brain Res. 1700, 190–198. doi: 10.1016/j.brainres.2018.09.002

Schambra, H. M., Ogden, R. T., Martínez-Hernández, I. E., Lin, X., Chang, Y. B., Rahman, A., et al. (2015). The reliability of repeated TMS measures in older adults and in patients with subacute and chronic stroke. Front. Cell. Neurosci. 9, 335. doi: 10.3389/fncel.2015.00335

Therrien-Blanchet, J.-M., Ferland, M. C., Rousseau, M.-A., Badri, M., Boucher, E., Merabtine, A., et al. (2022). Stability and test-retest reliability of neuronavigated TMS measures of corticospinal and intracortical excitability. Brain Res. 1794, 148057. doi: 10.1016/j.brainres.2022.148057

Vaseghi, B., Zoghi, M., and Jaberzadeh, S. (2015). Inter-pulse Interval Affects the Size of Single-pulse TMS-induced Motor Evoked Potentials: A Reliability Study. Basic Clin. Neurosci. 6, 44–51.
